# "Women Who Don't Give a Crap"

**DOI:** 10.1371/journal.pgen.1005736

**Published:** 2015-12-23

**Authors:** Holly Tabor

**Affiliations:** Department of Pediatrics, University of Washington, Seattle, Washington, United States of America

One of the often-bemoaned characteristics of modern life is the absence of leisure time due to chronic “busyness” and the constant connection afforded (or required) by our ever-present digital devices. As a result, time for nonessential reading is often in short supply, and we fiercely guard any minutes that we can steal from the other commitments of life. So when we do find time, what do we read? As the literary critic Harold Bloom said, “We read to find ourselves, more fully and strangely than we otherwise could hope to find.” Therefore, perhaps not surprisingly, when I get time, I read about women in science, past and present. Unfortunately, there are remarkably few books about the experiences of non-Nobel-Prize-winning female scientists, so I’ve had to look pretty hard to expand my reading list. In this Deep Reads article, I recommend four books about women and science that I read in 2015 (“When did you have time to read four books?” some will no doubt wonder). My hope is that *PLOS Genetics* readers, irrespective of gender or generation, will also find stories and ideas in these books that connect to their own professional and personal experiences (Fig 1).

**Fig 1:**
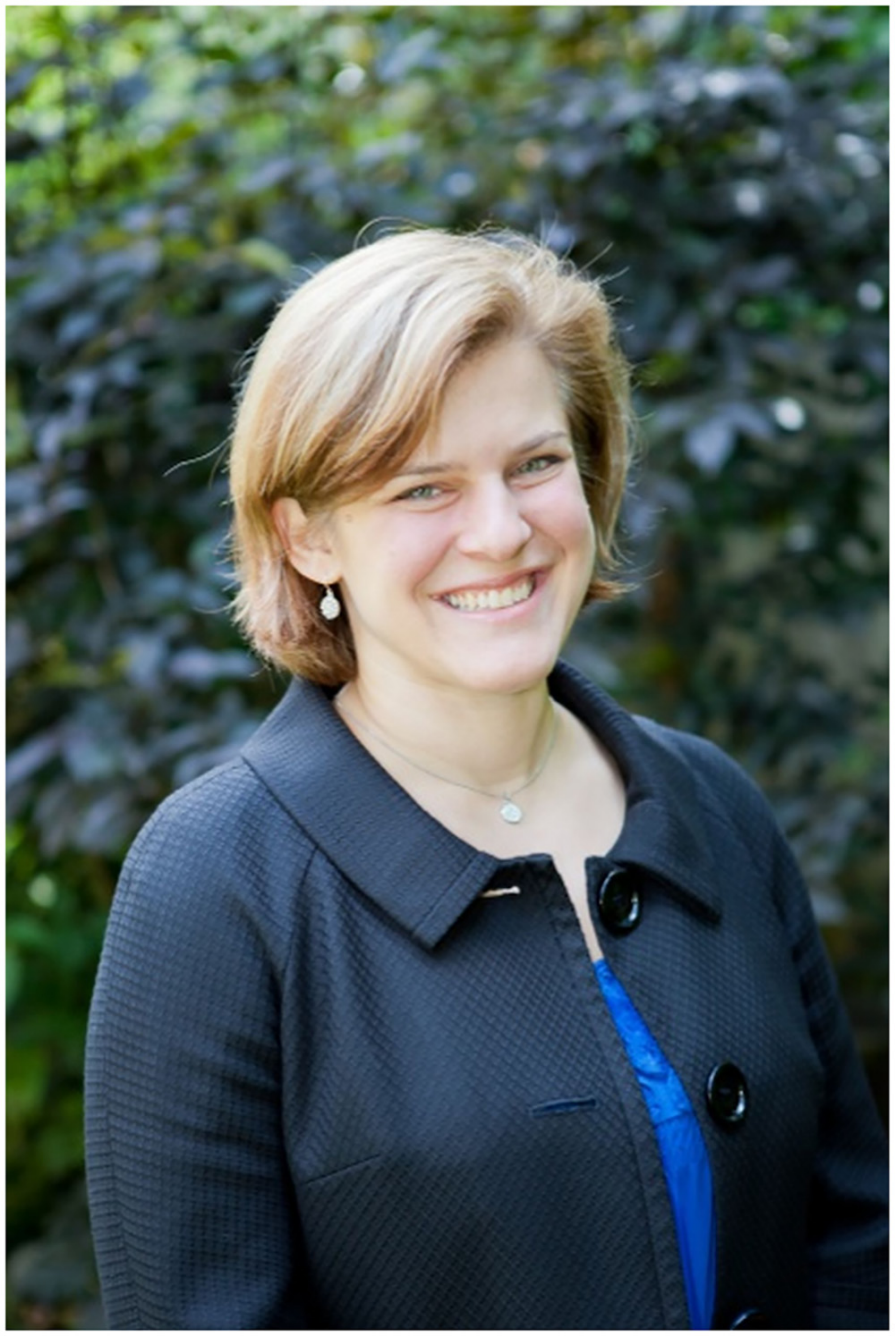
Holly Tabor. Image credit: Holly Tabor.

Profiles of great scientists chronicle not only their discoveries and career paths but also other dimensions of their lives and personalities. Too many profiles of great women scientists, however, have focused on their exceptionalism, too frequently treating the “subject’s sex as her most defining detail. She’s not just a great scientist, she’s a woman! And she’s also a wife and a mother, those roles get emphasized too!” [1]. In 2013, the New York Times obituary of the physicist Yvonne Brill raised controversy by opening with the sentence, “She made a mean beef stroganoff, followed her husband from job to job and took eight years off from work to raise three children,” deemphasizing her scientific career and accomplishments [2,3].

Science journalist Christine Aschwanden developed the “Finkbeiner Test” in response to the Brill obituary and this kind of journalistic bias. To pass the test, a profile of a woman scientist must not mention “the fact that she’s a woman, her husband’s job, her child care arrangements, how she nurtures her underlings, how she was taken aback by the competitiveness in her field, how she’s such a role model for other women, or how she’s ‘the first woman to…’” [4]. Alternatively, authors are encouraged to take what they write about a female subject and flip it around as if it were being said about a male, and if it sounds ridiculous, it doesn’t belong in the story [4].

While it is true that Brill’s obituary is an example of an absurdly skewed focus on women scientists as women first and scientists second, I think that it would be unfortunate and counterproductive to remove all references to other aspects of a scientist’s life, female or male, from an obituary or profile. There is an interesting and productive way to describe how a person, especially a woman, balanced myriad and competing expectations and obligations, especially social and institutional barriers and attitudes. This is the nuance of good biography and narrative, interwoven with social history, and to surgically remove certain aspects would be to tell an incomplete story. By examining the lives and struggles of successful women scientists in the past, we can learn about what needs to be done and what works to make more women successful in science in the future.

In *Headstrong*: *52 Women Who Changed Science and the World*, Rachel Swaby takes an intentionally different approach. She profiles 52 women scientists across a range of disciplines, including nine women in genetics and development. Swaby only includes women who have already died, acknowledging that this limits the diversity of her subjects. Nonetheless, their stories share common themes across disciplines and time: adventurousness and passion for science; persistence and perseverance in the face of obstacles; the influence of a supportive parent, mentor, collaborator, or spouse; battles over credit or even the grudging realization that credit for discoveries would likely never be forthcoming; reinvention and recovery from setbacks; creativity and innovation in dealing with a lack of title, resources, and lab space; and research on potentially unpopular topics for which the competition was less intense.

The short, three- to five-page profiles are introductory and include only a few narrative highlights, thereby missing some of the subtlety and nuance that would exist in a longer biography or even a dedicated article, which each of these women surely deserves (and a small number have). The *PLOS Genetics* reader will likely have heard of some of the women and not of others, but even the familiar scientists are presented with unusual stories or perspectives. *Headstrong* is an easy and fun read about a fascinating and tenacious group of women. It is an encouraging reminder of the largely untold legacy of women in science and their supporters, and it provides a connection between some of their challenges and those of the present day.

A more in-depth biography of one 20th century woman scientist and leader is *Mary Ingraham Bunting*: *Her Two Lives* by Elaine Yaffe. The book recounts the multiple stages of the life of Dr. “Polly” Bunting: from early bacteriologist, to stay-at-home mother and occasional researcher and teacher, to sudden widow and single mother, to Dean of Douglass College (the women’s college of Rutgers University), to President of Radcliffe College, to founder of the Bunting Institute, and, finally, to the first woman on the Atomic Energy Commission.

Bunting’s early life and young adulthood were characterized by the pursuit of a career in research science at a time when there were still significant institutional barriers to training, employment, and funding for women. Yaffe writes, “It was always her temperament to look on the positive side of things and to ignore unpleasantness,” and this attitude served her well throughout her life. The book is filled with examples demonstrating her improvisation and common sense, her juggling of time slots and commitments at work and at home, her efforts to break down complex problems into their component parts, and her patient efforts at brokering compromise and defying expectations.

Of particular interest is the description of Bunting’s invitation in 1946 to the Cold Spring Harbor Laboratory Symposium, organized by Salvador Luria, to present her pre-war, pre-motherhood research on color mutations of *Serratia* as an exceptional example of bacterial genetics [5]. Even though Bunting had been out of the lab for five years, Luria insisted on her coming, stating, “We simply cannot hold the Symposium without her.” Bunting recalled that since the men in science had paused their research for war work, “I wasn’t as far behind as I might have been.”

Bunting wrote the paper for the symposium with the support of a babysitter every Wednesday night, and her husband Henry took off several weeks in June so that she could focus on the work. She faced other challenges: during this time period, she also contracted German measles, and the family had to sleep in a 10-by-14-foot goat house on their property while their home was under construction. At the conference, Bunting was the only woman among the 26 invited presenters. While there, she networked with other participants to coin the phrase “microbial genetics” to describe the new and emerging field. The experience, combined with a stint of teaching bacteriology at Wellesley College, “reminded her how intensely she loved science and how hard it was to combine research with caring for small children.”

It would be years until Bunting returned to research part-time, and almost nine years until her husband died unexpectedly, forcing her to seek full-time work and shift to focus on the college education of women. But these early experiences made her acutely aware, even decades later, of the unique challenges faced by women who hoped to combine raising a family with the demands of a career in science. She dedicated much of her work later in life to developing innovative approaches to helping women transition back into the workplace after child-bearing and -rearing and to the gradual dissipation of the “climate of unexpectation” for women.

The third book, *The Only Woman in the Room*: *Why Science is Still a Boys’ Club* by Eileen Pollack, rose out of the author’s experience as one of the first two women to major in physics at Yale in the 1970s [6]. The first part of the book is a memoir, with Pollack recounting her early passion for science and dreams of becoming a theoretical astrophysicist. She describes the steps and missteps, bright moments, and, more frequently, isolation of her years at Yale as a true anomaly: a woman majoring in physics. Pollack describes, in sometimes excruciating detail, the battle to overcome the external lack of institutional support and encouragement as well as her own internal insecurities in a male-dominated field—a battle that she gave up when she decided to pursue a career in writing instead of going to graduate school in science.

The most compelling part of the book, however, is the second half, based on six years of Pollack investigating what has and hasn’t changed in science for women in the intervening years. She interviewed former teachers and classmates, current professors (male and female), and women science graduate students and post-docs. I was interested in her discussion of the subtle skills required for success, now that women in science are no longer anomalies. Specifically, Pollack provides an excellent examination of current tensions around expectations and both discouragement and encouragement from mentors and society.

Dr. Meg Urry, the first female Chair of the Physics Department at Yale, shared with Pollack her opinions about the role of confidence, or a confidence deficit, in scientific success for women. She observed that women are frequently socially conditioned to be less confident:

Over and over, Meg has seen women with above-average abilities drop out of science, while “more men of mediocre talent are determined to prevail…and they do.” It would be one thing if the weed-out system worked, she says. Then, even if the system weren’t kind, you could defend it. “But it just isn’t true. Lots of mediocre people have shitloads of confidence, and there are many brilliant people who have no confidence.” Even if you don’t start out being confident, “you can grow the boldness you need to be a good scientist.”

But how does one bolster or nurture such confidence and boldness? Pollack recounts attending a picnic for the Yale Physics and Astronomy Departments and a conversation with four young women post-docs. They recount how they have depended on the support of each other through the challenges of their training: “If there are enough women in your class, you can help each other get through.” Pollack asked them how they managed to persist (so far) in the pursuit of their dreams of a scientific career when so many others have failed. Their response: “Oh, that’s easy…we’re the women who don’t give a crap…about what people expect us to do. Or not do.” They continued to describe how success, for them, was characterized by a willingness to ignore the unwritten expectations of their field, of their contemporaries, and of society, about what it takes to be a successful woman scientist. While still early in their careers, these post-docs articulated and demonstrated some of the same perseverance and thick-skinned focus as their predecessors did in *Headstrong*, as well as Bunting’s predisposition to optimism in *Her Two Lives*.

Pollack also describes the research of Jo Handelsman, published in 2012, that demonstrated how implicit bias still exerts a pervasive influence on how men and women in science are perceived and evaluated. Her team sent curriculum vitae (CVs) to 127 professors of both genders: half received a CV from a man named “John,” and the other half received one from a woman named “Jennifer;” in all other respects, the CVs were identical. Each faculty member rated the candidates on numerous traits including competence, hireability, and likability. The results were striking: irrespective of the respondents’ age, gender, area of specialization, or seniority, the male candidate was rated at least half a point higher in all areas except likability, and the male candidate was offered an average starting salary of US$30,238, compared to the female candidate’s starting salary of US$26,308. The message, says Handelsman, is that implicit bias against women in science is serious, measurable, and so pervasive that it transcends gender, age, and discipline [7].

This is potentially discouraging: many of the biases demonstrated in Handelsman’s work are implicit, rather than explicit, and therefore harder to identify and mitigate. In the fourth book I am recommending, *Blindspot*: *Hidden Biases of Good People*, Mahzarin Banaji and Anthony Greenwald describe their extensive research with a tool designed specifically to detect and help combat implicit biases. The Implicit Association Tool (IAT) is a test that reveals the degree to which our unconscious biases, or “mindbugs,” defined as “imagined habits of thought that lead to errors in how we perceive, remember, reason and make decisions,” affect our perceptions. The authors argue that even if “good people” do not have conscious preferences or biases about many things (gender, race, disability, etc.), they still have empirically observable unconscious biases that influence their attitudes and actions. Even if you don’t read the book, I highly recommend taking some of the IATs available online at https://implicit.harvard.edu/implicit/takeatest.html. I think it is impossible not to be surprised and impressed by the results.

Banaji and Greenwald describe a study assessing IAT-measured gender-science stereotypes from 34 countries. Their data showed that the national gender differences in eighth-grade boys’ and girls’ math and science achievements was directly correlated with stronger *science = male* stereotypes: the greater the stereotype, the greater the gender performance disparity. This, combined with the dramatic decrease in the differences between boys’ and girls’ math and science performances over time, has helped to deflate the ever-persistent stereotype that boys have genetic advantages over girls in these fields and subjects.

So, what can we do to combat such pervasive mindbugs and biases? The final chapter of the book describes some empirically tested approaches. The authors caution, however, that implicit biases are likely elastic, and, while successful interventions can result in meaningful change, “they will require reapplication prior to each occasion on which one wishes them to be in effect.” The goal should be outsmarting implicit biases, rather than eradicating them.

I was especially intrigued by their discussion of self-undermining mindbugs, specifically the observation that not just men but also many women have automatic strong associations of the type *female = family* and *male = career*. Banaji and Greenwald write, “There is no reason to doubt that the mindbugs we direct toward ourselves are every bit as durable as those we direct towards others.” This is the kind of bias that Pollack described experiencing while at Yale in the 1970s and that many of the current students, post-docs, and faculty members that she interviewed worry still persists today.

Awareness of this unconscious self-undermining is a first step, and, perhaps, even more important when it occurs in the context of a social and institutional climate that reinforces it. However, it is not enough. Banaji and Greenwald describe some empirically proven institutional approaches to minimize this bias among women, including having women college students take math courses taught by female faculty and adding typically feminine décor to computer science classrooms. Telling women to just “believe in themselves” or not to “give a crap” and ignore their own implicit biases as well as those of others is clearly not adequate: institutions and the scientific community itself must find approaches to mitigate and outsmart all of our ongoing implicit biases in this area, all the more now that many explicit biases have been eliminated or diminished.

I hope that in the next few years there will be a proliferation of more detailed and nuanced biographies of “scientists who happen to be women,” including more recent stories. Ideally, such biographies would capture the rich depth of their narratives, including their scientific journeys and discoveries, as well as their experiences with the sociology of science, including perceptions of their work and roles as women in science. But I also hope that they include details about how they navigated other aspects of their lives, including partnership, parenthood, and other interests. Furthermore, I hope that additional research will be done documenting external and internal barriers and biases around women in science and the identification of more validated strategies to mitigate them.

## Information about the Books

Swaby R (2015) Headstrong: 52 Women Who Changed Science and the World. New York: Broadway Books.

Yaffe E (2005) Mary Ingraham Bunting: Her Two Lives. Savannah: Frederic C Beil.

Pollack E (2015) The Only Woman in the Room: Why Science is Still a Boys’ Club. Boston: Beacon Press.

Banaji MR, Greenwald AG (2013) Blindspot: Hidden Biases of Good People. New York: Delacorte Press (Available in paperback in August 2016).
